# The effectiveness of group-based pelvic floor muscle training in preventing and treating urinary incontinence for antenatal and postnatal women: a systematic review

**DOI:** 10.1007/s00192-021-04960-2

**Published:** 2021-08-28

**Authors:** Xiaowei Yang, Aixia Zhang, Lynn Sayer, Sam Bassett, Sue Woodward

**Affiliations:** 1 King’s College London, Department of Florence Nightingale Faculty of Nursing, Midwifery and Palliative Care, London, UK; 2 Nanjing Vocational Health College, Department of Clinical Teaching and Research Group, Nanjing, China; 3 Nanjing Maternity and Child Health Care Hospital, Nursing Department, Nanjing, China

**Keywords:** Antenatal, Group-based intervention, Plevic floor muscle training, Postnatal, Urinary incontinence

## Abstract

**Introduction and hypothesis:**

Urinary incontinence (UI) is prevalent in antenatal and postnatal women. Pelvic floor muscle training (PFMT) is the first-line treatment for UI. Group-based PFMT provides a way for professionals to deliver this intervention to more women who need to prevent and/or treat UI. This review aims to (1) assess the effectiveness of group-based PFMT in preventing and treating UI in antenatal and postnatal women and (2) explore the characteristics of group-based intervention and factors which had an impact on the success of group-based PFMT.

**Methods:**

Randomized controlled trials (RCTs) were included in this review. A comprehensive search was conducted in PubMed, Embase, Medline, PsycINFO, Maternity and Infant Care Database, CINAHL, Chinese Biomedical Literature Database, China National Knowledge Infrastructure, VIP Database and Wanfang Database. The overall quality was assessed using Grading of Recommendations, Assessment, Development and Evaluations (GRADE). RCTs which included pregnant and/or postnatal women with or without UI investigating the effectiveness of group-based PFMT were included.

**Results:**

Five RCTs were included in this review. The overall quality of the results of the included studies was low. Delivering group-based PFMT during pregnancy significantly reduced the prevalence of UI in both the pregnant period [risk ratio (RR) = 0.67, 95% confidence interval (CI) 0.57 to 0.80, *P* < 0.00001] and the postnatal period [RR = 0.66, 95% CI 0.52 to 0.84, *P* = 0.0008]. Only one RCT delivered group-based PFMT during the postnatal period.

**Conclusion:**

Evidence of weak quality supports the effectiveness of undertaking group-based PFMT in pregnancy to prevent UI during pregnancy and the postnatal period. No evidence showed the effectiveness of undertaking group-based PFMT in the postnatal period.

**Supplementary Information:**

The online version contains supplementary material available at 10.1007/s00192-021-04960-2.

## Introduction

The International Continence Society defines urinary incontinence as ‘a complaint of involuntary loss of urine’ [1]. Urinary incontinence (UI) is a common and costly problem affecting women from all age groups worldwide [2]. Millions of people worldwide are affected by urinary incontinence, and the reported prevalence in women varies from 9.3% to 67.1% [3]. However, it is also known that many women with symptoms of urinary incontinence under-report because of social embarrassment [4]. The cost of diagnosing and treating urinary incontinence is high, and it has many adverse effects on social activities, physical exercises and sexual relationships, although it does not endanger the lives of patients [5, 6].

Pregnancy and vaginal delivery are known to be associated with an increased risk of female urinary incontinence [7, 8]. There are many established risk factors for urinary incontinence during pregnancy and childbirth including increased abdominal pressure from the enlarging uterus, pressure on the pelvic floor muscles from the fetus and damage to the innervation of pelvic floor muscles during vaginal delivery [9, 10].

Multiple treatment options are provided to reduce the severity of urinary incontinence and to improve the quality of life of patients with urinary incontinence. For example, conservative behavioral interventions such as lifestyle modification, biofeedback treatment, vaginal cones as well as pelvic floor muscle training (PFMT) through to invasive surgery have been used. Pelvic floor muscle training as a conservative intervention was popularized by Arnold Kegel, so it is also known as the Kegel motion [11]. The National Institute for Health and Care Excellence (NICE 2015) recommends pelvic floor muscle training as a first-line conservative treatment for stress urinary incontinence (SUI) and any other type of UI. Although the knowledge and practice of PFMT in different populations may vary, women’s knowledge and practice of PFMT is still poor [12, 13].

Although the effectiveness of PFMT has been demonstrated [14], PFMT intervention is often not implemented well in clinical environments. Reasons for lack of implementation include the lack of healthcare professionals who can provide one-to-one training and lack of financial support [15, 16]. Group-based intervention is acknowledged as a useful tool in the field of health promotion, and it provides an economical and potentially scalable way to implement PFMT [17]. Additionally, this type of intervention is reported to increase individuals’ adherence and motivation by gaining peer support from the other participants in the group, which may have a positive impact on encouraging active self-management in the long term [18]. Group-based intervention has been applied in implementing pelvic floor muscle training and found to be effective in preventing and/or treating UI in women [19]. However, there is no systematic review assessing the effectiveness of group-based PFMT in preventing and/or treating UI in antenatal and postnatal women.

Based on the above considerations, this systematic review aims to answer the following research questions:

1. Is group-based delivery of PFMT effective in preventing and treating urinary incontinence in antenatal and postnatal women?
2. What are the potential barriers to and facilitating characteristics of group-based PFMT that influence the success of the group-based intervention?

## Materials and methods

### Design and registration

This systematic review was prepared according to the Preferred Reporting Items for Systematic Review and Meta-Analyses (PRISMA) [20, 21] and was registered prospectively in the PROSPERO database under protocol CRD42019135242.

### Search strategy

A systematic literature search was conducted in ten databases: PubMed, Embase, Medline, PsycINFO, Maternity and Infant Care Database, CINAHL, Chinese Biomedical Literature Database, China National Knowledge Infrastructure, VIP Database and Wanfang Database (From database inception to May 2021). The search used both Medical Subject Headings (MeSH) and free-text synonyms for the terms: pelvic floor muscle training, group therapy and urinary incontinence. An example search protocol is presented in supplementary material 1. The language of the literature was limited to English and Chinese. This systematic review was reported using the PRISMA flow diagram [22].

#### Eligibility criteria

The following eligibility criteria were defined:

1. Design and publication types: Randomized controlled trials (RCTs). Only studies published in peer-reviewed journals were included.
2. Type of participants: Pregnant and/or postnatal adult women (≥ 18 years old) with or without urinary incontinence. No limitations were set on the severity of urinary incontinence. The included postnatal period was set to no later than 6 months, according to the postpartum period defined by Romano et al. [23].
3. Type of intervention: PFMT delivered in a group format of any frequency or regimen and supervised by a registered health professional. The group-based intervention of PFMT can be delivered in any format including online supervision, face-to-face instruction or a combination of methods. Literature was excluded if the intervention of PFMT was combined with other treatments, for example, PFMT combined with electrical stimulation treatment or electromyographic biofeedback.
4. Types of comparison conditions: PFMT delivered by individualized supervision, standard care or usual care, which may include verbal instructions on PFMT or provide a leaflet on how to contract the pelvic floor muscles.
5. Type of outcome measures: Studies reporting any change in continence status, however measured. According to the recommendation of the International Continence Society (ICS), outcome measures can be selected from five categories [24]: (1) the patient’s observation, such as the perception of cure and improvement, (2) objective measures such as urine loss assessed by pad test or bladder diary, (3) clinician’s observation, for example, pelvic floor muscle activity or electromyography, (4) quality of life, which is normally assessed by specifically designed scales, and (5) socioeconomic measures. All the outcome measures from the above categories were eligible for the systematic review.

### Data extraction

A standardized form was used to extract the data from the included studies including study design, author, year of publication and country of study; participants’ characteristics (including age range, sample size, eligibility criteria and pregnancy status); interventions given including duration of the study, number of sessions and follow-up points; key results from data analysis; limitations and potential confounders of the studies mentioned by the author; potential barriers and facilitators of delivering group-based PFMT. If the information described in the study was insufficient, the authors were contacted through e-mail.

### Quality assessment

The Cochrane risk of bias tool was used to assess the quality of included randomized controlled trials. The assessment criteria included random sequence generation, allocation concealment, blinding of participants and personnel, blinding of outcome assessment, incomplete outcome data, selective reporting and other bias [25]. Each study was assessed by the above criteria and was rated low risk, high risk or unclear risk for each factor.

The overall quality of evidence was assessed by using the Grading of Recommendations Assessment, Development and Evaluation (GRADE) approach with overall quality of the evidence ranged from very low to high. The assessment criteria included the risk of bias, inconsistency, indirectness, imprecision and publication bias [26]. Two researchers (YXW and ZAX) independently assessed the quality of included trials and the overall quality of evidence.

### Data synthesis

To meet the review objectives, the data of the effectiveness of the intervention, outcome measurements and potential barriers and facilitating factors influencing the success of the group-based intervention were extracted and synthesized. Review Manager (RevMan 5.3) was used to analyze the quantitative data. For each outcome, relative risk (RR) or differences in means were pooled in the meta-analysis where possible. Heterogeneity across the included studies was assessed using I^2^ (between 30% to 60% may represent moderate heterogeneity, between 50% to 90% may represent substantial heterogeneity, 75% to 100% may be considerable heterogeneity) [27]. If the I^2^ was > 50%, random effects models were used, while if the I^2^ was < 50%, fixed effects models were used to calculate pooled RRs and 95% CIs. Tests for publication bias were planned, but not performed because an insufficient number of studies was available. The potential barriers and facilitating factors identified in the studies were extracted and included in the results and discussion providing information for future research.

## Results

### Study selection

**Fig. 1:**
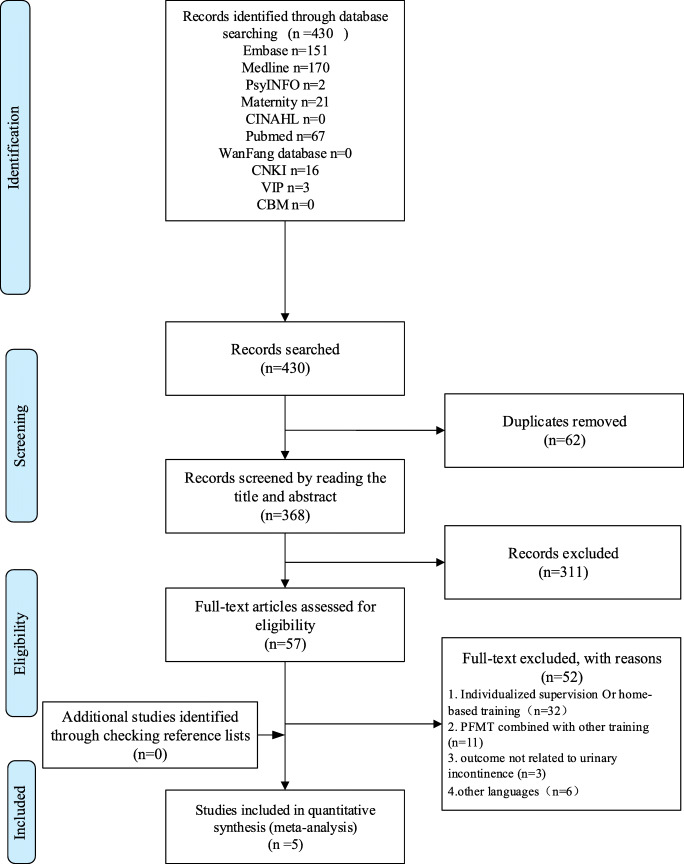
Study flow diagram

Using the search strategy, 430 articles were identified. Fifth-seven studies were potentially eligible for inclusion after the title and abstract screening, and five randomized controlled trials were finally included in this systematic review [28–32]. The selection process is shown in Fig. 1. Two studies were conducted in Norway [28, 31], and one each in the UK [29], Thailand [30] and China [32].

### Study characteristics

The general description of the included RCTs is presented in Table 1 and Table 2. There were 1132 participants included in the analysis in this systematic review. The participants in the included studies were pregnant and/or postpartum women having or not having complaints of urinary incontinence. Sample size varied between 70 [30] and 301 women [31]. Intervention duration ranged from 6 weeks [30] to 4 months [29], and the follow-up assessment lasted up to 6 months after delivery [30, 33]. Women in the pelvic floor muscle training group were instructed by physiotherapists or midwives in groups with intensive pelvic floor muscle exercises. Women in the control groups received either usual care, which may include information on PFMT, or no further instructions on these exercises.

**Table 1:** Characteristics of the PFMT regimen

**Study reference**	**Duration**	**Supervision frequency**	**Session number**	**Session duration**	**Expertise**	**Home exercises**	**Home exercises frequency**	**Support**	**How group intervention was defined**	**How adherence was recorded**
Bussara Sangsawang 2016	6 weeks	Biweekly	3	45 min	Midwife	Yes	Twice per day, at least 5 days	1. Make an appointment to remind about time and date 1 day before each session 2. A specially designed 25-page PFME handbook was provided	Four–five participants in a group	Did not state clearly in the study
S Morkved 2003	12 weeks from 20 to 32 pregnancy weeks	Weekly	12	60 min	Physiotherapist	Yes	Twice a day	Not provided	10–15 participants in a group	A training diary at home and group session adherence
Linda Mason 2010	4 months from 20 to 36 pregnancy weeks	Monthly	4	45 min	Physiotherapist	Yes	Twice a day	1. A reminder of the time and date of the class was sent to women a week before each session2. Class was held early evening	Participants attended a group session	Did not state clearly in the study
Gunvor Hilde 2013	16 weeks from 6 weeks after delivery	Weekly	16	Not provided	Physical therapist	Yes	Not provided	1. Customary leaflet2. Initial instruction on how to contract correctly	Group training session	A training diary at home and group session adherence

**Table 2:** Characteristics of included studies which assessed the effectiveness of PFMT during pregnant period

**Study characteristics**	**Participant characteristics**	**Intervention description**	**Comparator**	**Outcome measures**	**Results**	**Strength and limitation**
Sangsawang 2016; RCT; control group: *n* = 35 Intervention group: *n* = 35 Thailand; prevention study	Primigravid women without UI; gestational ages of 20–30 weeks; age range: 18–43 years old	** before intervention:** the participants must be ascertained to exercise the correct muscle by using stop-test; **PFMT protocol:** 20 sets of PFME exercises twice a day; at least 5 days per week during the whole 6 weeks, in different positions; each set of PFME includes one slow contraction (strong contraction for 10 s), followed by one fast contraction (briefly contracting and relax the muscle rapidly)	Regular prenatal care	Primary outcome: self-reported SUI; secondary outcome: severity of SUI (frequency, amount of urine leakage and visual analogue scale)	Significantly fewer women reported SUI in the intervention group at 38 weeks’ gestational age	Limitation: no objective indicators were assessed
Linda Mason 2010; RCT Control group: *n* = 145 Intervention group: *n* = 141; UK; prevention study	Nulliparous pregnant women; no symptoms of SUI; around 20th gestational week; age range: 17–41 years old	**Before intervention:** confirm women could do correct PFMT by digital assessment; **PFMT protocol:** near maximal muscle contraction held for 6 to 8 s, followed by three or four fast contractions in different positions; ** home exercises:** 8–12 maximal contractions repeated twice a day; ** used Bo’s protocol,** which was employed by three studies included in this review	Usual care and instruction in PFME; the ‘usual’ instruction ranged from occasional one-to-one exercising with an instructor, through a leaflet, through a brief reminder to nothing at all [43]	Bristol Female Lower Urinary Tract Symptoms Questionnaire (BFLUTS); Leicester Impact Scale (LIS); 3-day diary	The intervention group was significantly more likely to exercise their PFM; fewer episodes of incontinence and a lower score on the LIS in the intervention group, but not significant. The intervention group had lower total and average number of incontinence episodes (total: 57, mean: 1.06 ± 2.32) compared to the control group (total: 85, mean 0.77 ± 1.52); LIS score: control group (1.89 ± 3.08), intervention group (3.97 ± 3.80) (*P* > 0.05)	Limitation: low attendance of training sessions (the percentage of attendance was not provided in the study); the sample size only reached 70%, which is lower than the anticipated 80% power
Siv Morkved 2003; RCT Control group: *n* = 153 Intervention group: *n* = 148; Norway; prevention study	Nulliparous women; 18 gestational weeks; mean age: 28 years old in the intervention group; 26.9 years old in the control group	**Before intervention:** a correct contraction in both groups was confirmed by vaginal palpation and observation of inward movement of perineum; **PFMT protocol:** near maximal muscle contractions which were held for 6 to 8 s, followed by three to four fast contractions in different positions; **home exercises:** 8–12 equally intensive pelvic floor muscle contractions twice per day; ** used Bo’s protocol** which was employed by three studies included in this review	Customary information was given by their midwife or general practitioner; not discouraged from doing pelvic floor muscle exercise on their own	Primary outcome: self-report of UI; secondary outcome: episodes of urine leakage and whether the urinary leakage had changed in the home diary; pelvic floor muscle strength	Significantly fewer women reported UI and significantly higher pelvic floor muscle strength in the intervention group. The number of leakage episodes was significantly lower in the training group (25 of 148 versus 44 of 144, *P* = 0.014) at 36 weeks’ pregnancy, at 3 months postpartum 20 of 148 versus 34 of 144 were found, *P* = 0.049	Strength: high adherence to the training (81% women in the intervention group followed the training protocol)
Po-Chun Ko 2010; RCT Control group: *n* = 150 Intervention group: *n* = 150; China; mixed prevention and treatment study	Nulliparous women; 16–24 gestational weeks; mean age: 32 years old in the intervention group; 31 years old in the control group	**Before intervention:** confirm women could do correct PFMT by observation of inward movement of perineum during contraction; ** PFMT protocol:** eight contractions each held for 6 s, 2 min rest between three repetitions; **home exercises:** twice daily followed the PFMT protocol; **used Reilly’s protocol,** which was developed based on **Bo’s protocol**	Women received regular prenatal care and customary written postpartum instructions which did not include PFMT from the hospital, but they were not discouraged from performing PFMT on their own	Urogenotal Distress Inventory-6 (UDI-6); Incontinence Impact Questionnaire-7 (IIQ-7) and question of self-reported UI	Significantly lower total UDI-6 and IIQ-7 scores in the intervention group during late pregnancy and the postpartum period; self-reported UI was significantly lower in the intervention group; women who experienced vaginal delivery were more likely to develop UI than women who delivered by cesarean section at 3 days after delivery (38% versus 15%, *P* < 0.01) and 6 weeks after delivery (31% versus 13%, *P* = 0.01)	Limitation: no objective assessments and lack of data from long-term follow-up; strength: monitoring the adherence to the training program between sessions

### Methodology quality of included studies

**Fig. 2:**
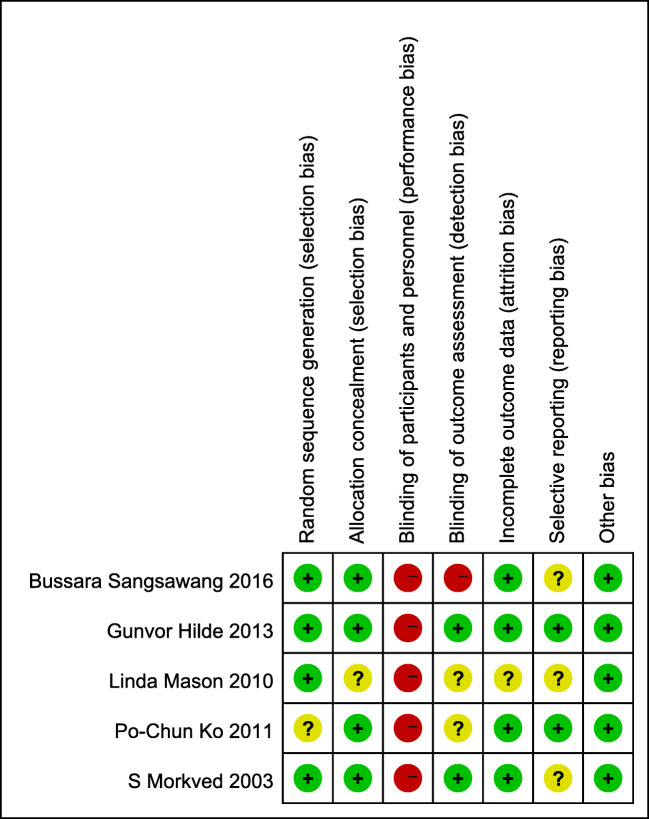
Risk of bias summary

The results of the risk of bias assessment are presented in Fig. 2. No study was assessed as low risk of bias for all categories. All the studies included in this review were at a high risk regarding blinding of participants and personnel as it is almost impossible to blind participants and therapists in physical therapy trials. Mason’s study only stated it was a single-blind trial; whether the outcome assessor was blind to the group allocation was unclear [29]. The outcome measurements which were rated at high risk were all patient-reported and were impossible to be blinded to the group allocation [30]. One study was judged to be unclear in incomplete outcome [29] because of high (33%) drop-out rates. The overall quality of the results of the included studies was assessed as low because of the high risk of bias and imprecision according to the GRADE analysis (supplementary material 2).

### Different protocols used in studies

Although pelvic floor muscle training has been recommended to prevent and treat UI for many years, there is no consensus over recommended frequency and required numbers of pelvic floor muscle contractions. Training regimen differed in the studies included in this systematic review. The detailed regimen employed in studies is presented below in Table 1, which highlights the variation in duration, supervision numbers, frequency and the numbers of participants in one group. Although different protocols were employed in the studies, an exercise protocol designed by Bo et al. [34] was used in three studies [28, 29, 31]. One further study followed the protocol from Reilly et al. [35], which was developed based on Bo’s protocol. However, two of these studies found no significant difference favoring the intervention group by using this protocol [28, 29], which contradicts the findings of the original study [34].

### The effectiveness of group-based PFMT delivered during pregnancy

Four randomized controlled trials on group-based PFMT commenced during pregnancy were identified [29–32]. Three of them assessed the prevention effect of group-based PFMT on UI in nulliparous women. The other was a mixed prevention and treatment study which included pregnant women without consideration of the UI status [32]. In these four studies, pregnant women (nulliparous women) were enrolled and started training from 16 [32] to 30 gestational weeks [30].

The first of these four studies was a prevention study conducted by Sangsawang et al. [30] using a 6-week supervised group-based PFMT program, including a specially designed handbook for the information of UI was delivered. The intervention group was compared to regular prenatal care which included postpartum instructions that did not include information on pelvic floor muscle exercise. The results of primary outcome, which was the self-report of presence of UI, were in favor of the intervention group with 27.3% of the participants (9/33) in the intervention group versus 53.3% of the participants (16/30) in the control group reporting UI (*P* = 0.018). The sencondary outcome measure was severity of stress urinary incontinence (SUI). The mean frequency of SUI was significantly lower in the intervention group (12.44 ± 5.27 versus 23.06 ± 5.72, *P* < 0.001). The perceived mean scores of SUI showed similar results (5.02 ± 0.89 versus 6.30 ± 1.20, *P* < 0.01).

The second prevention study by Mason et al. [29] compared group-based PFMT to usual care. The group-based PFMT program contained four sessions which lasted 45 min each. The prevalence of SUI was assessed by the Bristol Female Lower Urinary Tract Symptoms Questionnaire (BFLUTS). The exercise group did not appear to have significantly lower prevalence of UI compared with usual care at both 36 gestational weeks (40% in the intervention group and 53% in the control group, *p* = 0.138) and 3 months after delivery (33.8% in the intervention group and 41.3% in the control group, *p* = 0.397). Compared with usual care, the participants in the intervention group scored lower on the Leicester Impact Scale (LIS) and had lower total and average number of incontinence episodes at both 36 gestational weeks and 3 months after delivery, but the differences were not statistically significant.

The third prevention study provided a 12-week intensive pelvic floor muscle training program to nulliparous women, which was compared with participants who received the customary information [31]. It was found that fewer women in the training group reported UI during pregnancy (32% in the intervention group versus 48% in the control group, *P* = 0.007) and 3 months after delivery (20% in the intervention group versus 32% in the control group, *P* = 0.018), which was the primary outcome measure. Results of the secondary outcome measure, which was the pelvic floor muscle strength, was also in favor of the intervention group at 36 gestational weeks (39.9 cmH_2_O versus 34.4 cmH_2_O, *P* = 0.008) and 3 months after delivery (29.5 cmH_2_O versus 25.6 cmH_2_O, *P* = 0.048).

The last trial which delivered a group-based PFMT program during pregnancy was a mixed prevention and treatment study [32]. Women in the intervention group followed a group-based PFMT program consisting of 12 training sessions. The control group received regular prenatal care, which did not include PFMT. It was found that the intervention group had significantly lower total UDI-6 scores during 36 gestational weeks (3.44 ± 3.26 versus 4.66 ± 3.32, *P* < 0.01), 3 days after delivery (1.42 ± 2.04 versus 2.31 ± 2.16, *P* < 0.01), 6 weeks after delivery (0.81 ± 1.36 versus 1.54 ± 1.59, *P* < 0.01) and 6 months after delivery (0.35 ± 0.84 versus 0.86 ± 1.14, *P* < 0.01). The IIQ-7 score showed similar results during 36 gestational weeks (3.77 ± 6.01 versus 5.28 ± 5.61, *P* < 0.01), 6 weeks after delivery (1.73 ± 3.57 versus 2.86 ± 3.52, *P* < 0.01) and 6 months after delivery (0.77 ± 2.07 versus 1.56 ± 2.20, *P* < 0.01). Also, the prevalence of UI in the intervention group was significantly lower compared to the control group at 36 gestational weeks (34% in the intervention group versus 51% in the control group, *P* < 0.01).

**Fig. 3:**
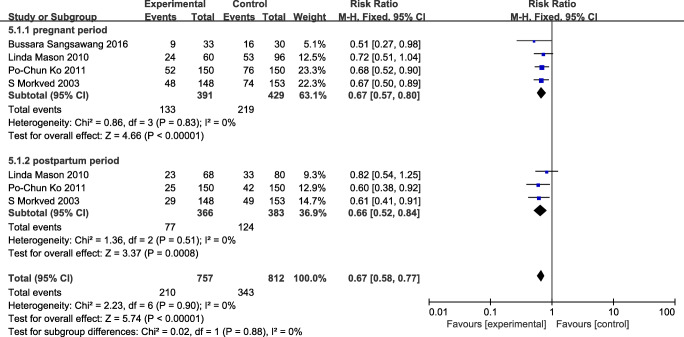
Effectiveness of doing group-based PFMT during pregnant period

In total, 957 participants were included in the analysis during pregnancy. Because of the different outcome measures employed by different trials, only self-reported UI was available to be pooled in a meta-analysis (Fig. 3). Three studies assessed the prevalence of UI in both pregnancy and the postnatal period [29, 31, 32], and all four studies assessed the the prevalence of UI in the pregnancy period. Data from the RCTs found that group-based PFMT significantly reduced the prevalence of UI versus the control group in both the pregnant period (risk ratio = 0.67, 95% CI 0.57 to 0.80, *P* < 0.0001, I^2^ = 0%) [29–31] and postnatal period (risk ratio = 0.66, 95% CI 0.52 to 0.84, *P* = 0.0008, I^2^ = 0%) [29, 31].

Overall, combined with the results of the meta-analysis, it was found that delivering PFMT during pregnancy in groups was effective in preventing UI in both pregnancy and the postnatal period by reducing the prevalence of UI. A detailed study description is presented in Table 2).

### Effectiveness of group-based PFMT after childbirth

Only one study that assessed the role of group-based PFMT on both prevention and treatment during the postpartum period was identified [28]. Hilde et al. [28] found group-based postpartum PFMT did not decrease UI prevalence 6 months after delivery in primiparous women. This was a mixed prevention and treatment trial which recruited primiparous women with and without UI. Hilde et al. [28] provided a 16-week training program which started from 6 to 8 weeks after delivery to participants and assessed self-reported UI, urine leakage, vaginal resting pressure, pelvic floor muscle strength and pelvic floor muscle endurance at 6 weeks and 6 months after delivery. It was found that there was no significant difference in all the outcome measures between the two groups at 6 weeks and 6 months after delivery (*P* > 0.05). The detailed study description is presented below (Table 3).

**Table 3:** Characteristics of included studies which assessed the effectiveness of PFMT during the postpartum period

**Study characteristics**	**Participant characteristics**	**Intervention description**	**Comparator**	**Outcome measures**	**Results**	**Strength and limitation**
Hilde 2013 RCT Control group: *n* = 87 Intervention group: *n* = 88 Norway Mixed prevention and treatment study	Singleton primiparous women who delivered vaginally after > 32 weeks gestation; mean age: 29.8 years old	**Before intervention:** a correct contraction was confirmed by observation and palpation **PFMT protocol:** 8–12 close to maximum contractions with each held for 6 to 8 s; followed by three or four fast contractions in different positions **Home exercises:** three sets of 8 to 12 contractions close to maximum **Used Bo’s protocol,** which was employed by three studies included in this review	No further intervention	Primary outcome: ICIQ-SF Secondary outcome: pad test; vaginal resting pressure; pelvic floor muscle strength; pelvic floor muscle endurance	No significant difference was found in self-reported UI between the two groups. 30 of 87 (34.5%) in the intervention group versus 34 of 88 (38.6%) in the control group reported UI at 6 months after delivery (*P* = 0.57); 23 of 87 (21.8%) in the intervention group versus 23 of 88 (26.1%) in the control group had positive pad test at 6 months after delivery (*P* = 0.69); mean differences at the intervention test were 1.3 cmH_2_O for vaginal resting pressure (*P* = 0.257), 3.3 cmH_2_O for floor muscle strength (*P* = 0.172) and 29.8 cmH_2_O for endurance (*P* = 0.148)	Limitation: drop out imbalance between two groups (12 women in the intervention group dropped out by only three women dropped out from the control group).

### Potential barriers influencing group-based intervention

A high drop-out rate and imbalance in the intervention group were found by Hilde et al. [28] (20% attrition in the intervention group compared with 5% in the control group). Mason et al. [29] recognized that it would be time-consuming for pregnant participants who worked until the end of their pregnancy, so the they offered early evening classes to accommodate the women who had to work during the daytime; however, the class attendance was reportedly still lower than anticipated (no data on attendance were provided).

### Facilitating factors influencing group-based intervention

Regarding the facilitating factors influencing group-based intervention, four studies mentioned the adherence of the participant to the exercise regimen [28–30, 32]. Close supervision by an experienced clinician, which contributed to strong adherence to and participants’ motivation for the treatment, was recommended by all the trials included in this systematic review. Sangsawang et al. [30] and Mason et al. [29] tried some useful ways to improve the adherence of women to the PFMT, for example, giving women verbal instructions in combination with a PFMT handbook as well as supervision in hospital [30], holding classes at a location where parking was available [29] and sending a reminder 1 week before class [29, 30]. However, as this support was provided as a part of the PFMT program, the effectiveness of this support alone cannot be analyzed separately. Also, Sangsawang et al. [30] found that an intensive PFMT regimen could improve the compliance of participants with doing more exercises during the intervention period, while in Ko et al.’s study, the importance of adherence to the training protocol was emphasized by the physiotherapists who led the training groups [32].

## Discussion

This is the first review to our knowledge to explore the effectiveness of group-based pelvic floor muscle training among pregnant and postnatal women. In this review, group-based antenatal PFMT was found to be effective in reducing the prevalence of UI, and this latent effect can persist up to 6 months after delivery compared with usual care. This finding was consistent with a previous Cochrane review which was conducted to assess the effects of PFMT on preventing or treating UI and fecal incontinence in pregnant or postnatal women [14]. For the group-based postpartum PFMT, only one study was identified. The study was a mixed prevention and treatment study and found that group-based postpartum PFMT did not decrease the prevalence of UI 6 months after delivery [28].

In this review, three studies clearly stated the primary and secondary outcome measures in the methods [28, 30, 31]. The other two studies [29, 32] used multiple outcome measures without defining the primary and secondary outcome measures, which may have increased the risk of false-positive errors [36]. Self-reported UI was the only outcome measure used in all the studies included in this review, and only the results of self-reported UI could be pooled in a meta-analysis in this review. This outcome measure can be categorized as the patient’s observations according to the recommendation of ICS [37]. In the studies included in this review, several objective measures such as urine leakage and pelvic floor muscle strength were also used. If measurements can be selected from different domains including both objective and subjective measures, the overall value of study results will be enhanced [37]. In addition, the significance of results from studies may vary by choosing different outcome measures and consequently impact the interpretation of the intervention effectiveness.

The meta-analysis showed that doing group-based PFMT during pregnancy can significantly reduce the prevalence of UI both during pregnancy and 6 months after delivery. However, this finding needs to be interpreted with caution because the sample size was relatively small, and one study did not reach the numbers of participants planned, which resulted in achieving 70% power rather than the 80% they aimed for [29]. The results from the postnatal study also need to be interpreted with caution because of the baseline and drop-out rate imbalance found in this study between the two groups [28].

Analyzing the effect of group-based PFMT on the prevention and treatment of UI is complex because the training regimens employed in the studies varied greatly. In addition, the details of the programs in some studies were poorly described [28, 31]. Indeed, the type of exercises, frequency of training, intensity of supervision and duration of the whole training have a great impact on effect size [38]. Although the regimens used in these trials differed, great homogeneity was found in the intensity and frequency of the training. Three trials included in this review followed the same PFMT regimen, which was proposed by Bo et al. [34]. The PFMT regimen was designed to increase both strength and endurance of skeletal muscles [39]. Bo et al. [34] found this PFMT program was effective in treating genuine stress incontinence. Similar positive results were found in Reilly’s study [35] and Morkved’s study [31]. Reilly et al. [35] provided the PFMT program individually to the participants on a monthly basis from 20 weeks of pregnancy. It was found that fewer women in the intervention group reported stress urinary incontinence in the study [35], and the protocol from Reilly was employed by one of the included studies [32]. Morved et al. [31] provided the PFMT program to healthy pregnant women and found that women in the intervention group had a reduction in self-reported UI and the number of episodes of urine leakage and an improvement in pelvic floor muscle strength. However, Hilde et al. [28] and Mason et al. [29] found no significant improvement in the intervention group when using the same PFMT regimen. The imbalance of drop-out rates in different groups [28], the imbalance between comparison groups on reported UI at baseline [28], insufficient sample size and low adherence to the program [29] may contribute to the non-significant research results.

Group-based training has been implemented in behavioral therapies and PFMT for many years as it is as effective as individualized training in community-dwelling women and requires less money and human resource compared to individual training [19, 40]. A recent systematic review which aimed to assess the most cost-effective way of providing PFMT to prevent or treat postpartum UI found that for women with UI after delivery, providing group-based PFMT for women during pregnancy seemed to be more efficient than individual PFMT [41].

However, only five RCTs were documented to assess the effectiveness of group-based PFMT in pregnant or postnatal women. In addition, the comparison group in the five studies was usual care or no further intervention. Whether group-based PFMT is as effective as individualized PFMT in pregnant or postnatal women is still unknown. Two studies in this review mentioned individualized PFMT may provide higher adherence to the training program [28, 29]. However, the authors did not state the reason and evidence for this viewpoint. Mason et al. [29] inferred this because no significant difference was documented in the study by using the same PFMT regimen which appeared to be successful in other studies [34]. However, the low response rate to the questionnaires, slow recruitment and low attendance at the exercise classes in the study indicated the participants may have had little interest in PFMT. The high drop-out rate and an insufficient number of participants were the main flaws in the study and also may be the main reason for the insignificant improvement in the intervention group [29]. Hilde’s [28] study mentioned that when studies included women with poor pelvic floor muscle function or severe UI, individually supervised training may be more successful than a class-based intervention, but the author did not provide the evidence for this viewpoint. One of the limitations of Hilde’s [28] study was the different dropout rates between groups, which may not be random. Twelve women dropped out from the intervention group compared to three women from the control group in the study. Hilde suggested that another reason for the insignificant results was that the study included a number of women with major levator ani defects. In addition, it was a mixed prevention and treatment trial, which could result in less effectiveness than studies only targeting either prevention or treatment of UI [28].

A Cochrane review found that PFMT had a positive effect in protecting healthy pregnant women from UI, and this effect could persist up to 6 months after delivery [14]. For postnatal women with UI after delivery, the participants were less likely to report UI compared to participants who received no treatment or usual care [42]. However, the Cochrane review aimed to compare the effect of PFMT on UI to usual care in antenatal or postnatal women no matter how the PFMT was delivered. Both studies which used individualized PFMT supervision and group-based PFMT supervision were included in the Cochrane review and were analyzed together. This review assessed the effect of group-based PFMT on UI against usual care. Our findings are consistent with the Cochrane review in pregnant women [42]. The conclusion for the postnatal women, however, was not consistent. One of the possible reasons was only one study assessed the effect of group-based PFMT after delivery, and this study included women with and without the symptom of UI, which means this was a mixed prevention and treatment study [28]. Therefore, trials on the effect of group-based PFMT in postnatal women still need to be further studied. Paiva et al. [19] published a systematic review to compare the effects of group-based PFMT with individual or home training in the treatment of UI. Ten studies with 927 women were identified in the review. It was found that PFMT was effective in improving the symptom of UI in incontinent women, and there was no significant difference between group training and individual training when PFMT was supervised by a physiotherapist, but group PFMT was more efficient in the treatment of UI than home training. Unfortunately, no studies included in Paiva’s review recruited pregnant or postnatal women [19], which is the population of interest in this review.

### Limitation of the study

First, the number of included studies was limited. Second, most studies included in this review were of poor methodological quality and did not report in line with the Consolidated Standards of Reporting Trials (CONSORT) statement. The sample size of most of the studies was relatively small, and in one trial, the sample size did not reach the power which the authors aimed for to detect significant differences between the intervention group and the control group [29]. Also, the outcome measures reported in some studies were incomplete to support further statistical analysis [28, 29].

### Recommendations for clinical practice

Group-based PFMT should be implemented during pregnancy to prevent UI during pregnancy and the postnatal period. Although the evidence of delivering group-based PFMT during pregnancy was of weak quality, it still provided a potential way of delivering PFMT to a larger population of pregnant women using a limited number of professionals.

## Conclusions

A limited number of studies was found to assess the effectiveness of group-based PFMT in pregnant or postnatal women. Despite the heterogeneity of the PFMT regimen and the great variety of outcome measures, compared to usual care, evidence of weak quality from the studies supports the effectiveness of doing group-based PFMT in the pregnant period to prevent UI during pregnancy and the postnatal period. While group-based interventions could provide an economical way to implement PFMT, well-designed randomized controlled trials with high methodological quality, adequate sample size, validated training protocols and outcome measures are needed to provide evidence of the effect of group-based PFMT in pregnant or postnatal women.

## Supplementary Information

ESM 1 (DOCX 13 kb)

ESM 2 (PDF 27 kb)

## Abbreviations

UI: Urinary incontinence
GRADE: Grading of Recommendations, Assessment, Development and Evaluations
PFMT: Pelvic floor muscle training
SUI: Stress urinary incontinence
RCT: Randomized controlled trial
PRISMA: Preferred Reporting Items for Systematic Review and Meta-Analyses
NICE: The National Institute for Health and Care Excellence
ICS: International Continence Society
ICIQ-SF: International Consultation on Incontinence Questionnaire-Short Form
BFLUTS: Bristol Female Lower Urinary Tract Symptoms Questionnaire
LIS: Leicester Impact Scale
CI: Confidence interval
RR: Relative risk
SD: Standard deviation
