# Supplementary material for: The effectiveness of group-based pelvic floor muscle training in preventing and treating urinary incontinence for antenatal and postnatal women: a systematic review
Source: Int Urogynecol J. 2021 Aug 28;33(6):1407–20. doi: 10.1007/s00192-021-04960-2 (PMC9206632; doi:10.1007/s00192-021-04960-2)
Supplement: Supplementary file 1 — (DOCX 13 kb) [file 192_2021_4960_MOESM1_ESM.docx]

| **Sample search strategy for Ovid Medline** |
| --- |
| 1 urinary incontinence.mp. or exp Urinary Incontinence/ |
| 2 exp Pelvic Floor/ or pelvic floor muscle training.mp. |
| 3 pelvic floor muscle exercise.mp. |
| 4 PFMT.mp. |
| 5 PFME.mp. |
| 6 pregnant.mp. or exp Pregnancy/ or exp Pregnant Women/ |
| 7 postpartum.mp. or exp Postpartum Period |
| 8 postnatal.mp. or exp Postnatal Care/ |
| 9 exp Prenatal Care/ or prenatal.mp. |
| 10 antenatal.mp. |
| 11 group.mp. or exp Peer Group/ or exp Group Practice/ |
| 12 group therapy.mp. |
| 13 group treatment.mp. |
| 14 group intervention.mp. |
| 15 2 or 3 or 4 or 5 |
| 16 6 or 7 or 8 or 9 or 10 |
| 17 11 or 12 or 13 or 14 |
| 18 1 and 15 and 16 and 17 |
