# Supplementary material for: The effectiveness of group-based pelvic floor muscle training in preventing and treating urinary incontinence for antenatal and postnatal women: a systematic review
Source: Int Urogynecol J. 2021 Aug 28;33(6):1407–20. doi: 10.1007/s00192-021-04960-2 (PMC9206632; doi:10.1007/s00192-021-04960-2)
Supplement: Supplementary file 2 — (PDF 27 kb) [file 192_2021_4960_MOESM2_ESM.pdf]

Group-based PFMT compared to ususal care for preventing or treating UI in pregnant or postpartum women

Bibliography: . PFMT for UI in pregnant or postpartum women. Cochrane Database of Systematic Reviews [Year], Issue [Issue].

Certainty assessment							Summary of findings				
Participants (studies) Follow up	Risk of bias	Inconsistency	Indirectness	Imprecision	Publication bias	Overall certainty of evidence	Study event rates (%)		Relative effect (95% CI)	Anticipated absolute effects	
							With ususal care	With group-based PFMT		Risk with ususal care	Risk difference with group-based PFMT
**self-reported UI (follow up: range 36 weeks to 6 months; assessed with: participant numbers)**											
1569 (4 RCTs)	serious [a]	not serious	not serious	serious [b]	none	⨁⨁◯◯ LOW	343/812 (42.2%)	210/757 (27.7%)	**RR 0.67** (0.58 to 0.77)	422 per 1,000	**139 fewer per 1,000** (from 177 fewer to 97 fewer)
**self-reported UI during pregnancy period (follow up: range 36 weeks to 38 weeks; assessed with: participant numbers)**											
820 (4 RCTs)	serious [a]	not serious	not serious	serious [b]	none	⨁⨁◯◯ LOW	219/429 (51.0%)	133/391 (34.0%)	**RR 0.67** (0.57 to 0.80)	510 per 1,000	**168 fewer per 1,000** (from 220 fewer to 102 fewer)
**self-reported UI during the postpartum period (follow up: range 3 months to 6 months; assessed with: participant numbers)**											
749 (3 RCTs)	serious [a]	not serious	not serious	serious [b]	none	⨁⨁◯◯ LOW	124/383 (32.4%)	77/366 (21.0%)	**RR 0.66** (0.52 to 0.84)	324 per 1,000	**110 fewer per 1,000** (from 155 fewer to 52 fewer)

CI: Confidence interval; **RR:** Risk ratio

Explanations

a. Unclear allocation concealment and blinding of outcome assessors
b. Insufficient sample size
